# Effects of Ellagic Acid and Berberine on Hind Limb Ischemia Reperfusion Injury: Pathways of Apoptosis, Pyroptosis, and Oxidative Stress

**DOI:** 10.3390/medicina61030451

**Published:** 2025-03-04

**Authors:** Esra Tekin, Ali Koray Kaya, Ayşegül Küçük, Mustafa Arslan, Abdullah Özer, Hüseyin Demirtaş, Şaban Cem Sezen, Gülay Kip

**Affiliations:** 1 Department of Physiology, Faculty of Medicine, Kutahya Health Sciences University, Kutahya 43020, Turkey; esra.tekin@ksbu.edu.tr (E.T.); alikoray.kaya@ksbu.edu.tr (A.K.K.); kucukaysegul@hotmail.com (A.K.); 2 Department of Anesthesiology and Reanimation, Faculty of Medicine, Gazi University, Ankara 06500, Turkey; gulaykip@yahoo.com; 3 Department of Cardiovascular Surgery, Faculty of Medicine, Gazi University, Ankara 06500, Turkey; dr-abdozer@hotmail.com (A.Ö.); drhuseyindemirtas@yahoo.com (H.D.); 4 Department of Histology and Embryology, Faculty of Medicine, Kırıkkale University, Kırıkkale 71450, Turkey; sezenscem@gmail.com

**Keywords:** hind limb ischemia–reperfusion injury, ellagic acid, berberine, apoptosis, pyroptosis, oxidative stress, rat

## Abstract

*Background and Objectives*: Hind limb ischemia–reperfusion (I/R) injury is a serious clinical condition that requires urgent treatment and develops as a result of a sudden decrease in blood flow in the extremity. Antioxidant combinations are frequently used in diseases today. This study aimed to investigate and compare the effectiveness of ellagic acid (EA) and berberine (BER), which are important antioxidants, and the combination on hind limb I/R injury to evaluate their therapeutic power. *Materials and Methods*: Thirty-five male Sprague Dawley rats were randomly divided into five groups: sham, I/R, EA+I/R, BER+I/R, and EA/BER+I/R. In the I/R procedure, the infrarenal abdominal aorta was clamped and reperfused for 2 h. EA (100 mg/kg, ip) and BER (200 mg/kg, ip) were administered in the 75th minute of ischemia. Oxidative stress markers (MDA, GSH, SOD, and CAT) and TNF-α were measured. Apoptosis (Bax, Bcl-2, and Cleaved caspase-3) and pyroptosis (Nrf2, NLRP3, and Gasdermin D) pathways were evaluated via Western blot. Muscle tissue was examined histopathologically by hematoxylin eosin staining. One-way ANOVA and post hoc LSD tests were applied for statistical analyses (*p* < 0.05). *Results*: Bax levels increased in the ischemia group and decreased with EA and BER (*p* < 0.05). Bcl-2 levels decreased in the ischemia group but increased with EA and BER (*p* < 0.05). The highest level of the Bax/Bcl-2 ratio was in the I/R group (*p* < 0.05). Cleaved caspase 3 was higher in the other groups compared to the sham group (*p* < 0.05). While Nrf2 decreased in the I/R group, NLRP3 and Gasdermin D increased; EA and BER normalized these levels (*p* < 0.05). In the histopathological analysis, a combination of EA and BER reduced damage (*p* < 0.05). TNF-α levels were similar between groups (*p* > 0.05). MDA levels were reduced by EA and BER, but GSH, SOD, and CAT levels were increased (*p* < 0.05). *Conclusions*: It was concluded that TNF-α levels depend on the degree and duration of inflammation and that no difference was found in relation to duration in this study. As a result, EA, BER, and their combination could be potential treatment agents on hind limb I/R injury with these positive effects.

## 1. Introduction

Hind limb ischemia–reperfusion (I/R) injury is a serious clinical condition based on a sudden decrease in blood flow in an extremity, and develops as a result of arterial occlusion, vascular injuries, vasculitis, and compartment syndrome [1]. The gold standard treatment for hind limb ischemia is emergency revascularization. Serious complications such as tissue necrosis, infection, and limb loss may develop [2]. The basis factors of this condition are oxidative stress, which occurs as a result of the imbalance between reactive oxygen species and antioxidant systems, inflammation, and cell death pathways [3,4].

Apoptosis is an important mechanism that ensures the controlled and programmed death of cells and maintains homeostasis [5]. During I/R injury, oxidative stress and inflammatory processes lead to the triggering of apoptosis [6]. The apoptotic process begins with the activation of intracellular death signals. One of the most important indicators in this process is caspase-3. Bax and Bcl-2 play crucial roles in the regulation of the apoptosis [7,8]. Pyroptosis is a programmed cell death that develops due to inflammation and plays a critical role in I/R injury [9]. This process begins with the activation of the NLRP3 inflammasome complex and continues with the activation of caspase-1. Gasdermin D (GSDMD), one of the important marker proteins of pyroptosis, is activated by caspase-1, creates pores in the cell membrane, and causes the release of inflammatory mediators [10]. Oxidative stress is known as one of the factors that triggers pyroptosis, and Nrf2 can limit the severity of pyroptosis via reducing oxidative stress [11]. Various antioxidant agents, like ellagic acid and berberine, are used to prevent oxidative stress and cell death pathways.

Ellagic acid (EA) is a polyphenolic compound naturally found in various fruits and vegetables, especially in foods such as pomegranates, strawberries, and walnuts. EA, an anti-inflammatory, antimicrobial, and antitumor agent, has a protective effect on tissues by reducing oxidative stress [12]. It is known for its positive effects on the liver, kidney, testis, and brain, and has a protective effect against I/R injury by suppressing inflammatory responses [13,14,15,16].

Berberine (BER), an antioxidant, anti-inflammatory, and antimicrobial agent, contributes to the protection of tissues by suppressing cellular death mechanisms [17,18]. BER, an alkaloid, is an active ingredient in many plants and has protective effects against I/R injury on the heart, brain, and kidney tissues [19,20,21].

Antioxidant combinations are widely used today. However, it is not known whether the combined forms are more effective than their separate forms or whether they cause negative results. Therefore, in this study, the effectiveness of EA and BER, known to have healing effects, was compared with their combination and investigated on hind limb I/R injury.

The current study aimed to evaluate and compare the effects of EA and BER on hind limb I/R injury via cell death pathways and oxidative stress.

## 2. Materials and Methods

### 2.1. Animals and Experimental Groups

**Figure 1:**
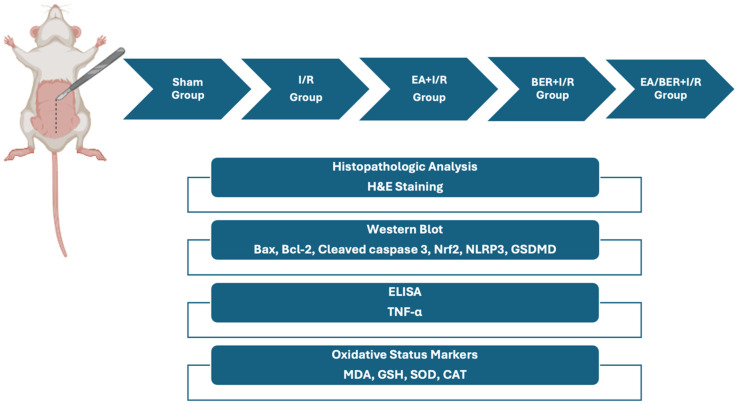
Graphical representation of the experimental method.

Thirty-five Sprague Dawley male rats were used and housed in the laboratory at 12 h in daylight and 12 h in darkness. Rats were randomly divided into 5 groups (n = 7): sham, I/R, EA+I/R, BER+I/R, and EA/BER+I/R. Rats were anesthetized with 45 mg/kg of Ketamine Hydrochloride and 5 mg/kg of Xylazine Hydrochloride intramuscularly. If necessary, maintenance anesthesia (20 mg/kg of ketamine) was applied for deep anesthesia. In the sham group, a midline laparotomy was performed, but no clamping was applied. Hind limb ischemia was performed for 2 h by clamping the infrarenal abdominal aorta, then 2 h of reperfusion was applied. To avoid hypovolemia, an hourly 3 mL/kg ip isotonic solution was administered. During I/R, the abdomen was covered with a gauze with isotonic solution. A 100 mg/kg ip EA (324683, Sigma-Aldrich, St. Louis, MO, USA) [14,22,23] and a 200 mg/kg ip BER (B3412, Sigma-Aldrich, St. Louis, MO, USA) [24] were administered as a single dose in the 75th minute of ischemia. Considering the half-lives of EA and BER and the articles in the literature, they were given once at the 75th minute of ischemia [24,25] (Figure 1).

At the end of the 2 h reperfusion period, the rats were sacrificed. After the application of 100 mg/kg of ketamine, intracardiac blood was taken and the sacrificial process was performed. Skeletal tissue samples were collected for Western blot, ELISA, and histopathologic analysis and stored under appropriate conditions until used. For each experiment, 7 samples from each group were used (n = 7). 

### 2.2. Histopathologic Analysis

Muscle tissue samples were immersed in 10% neutral-buffered formalin and fixed for 48 h for histopathological analysis. After fixation, tissue samples were routinely processed. Next, 4 μm thick sections were sliced from paraffin blocks using a microtome, and the sections were stained with hematoxylin and eosin (H&E), then images were obtained from a light microscope. The morphological properties of muscle samples were evaluated, and each parameter was scored according to the following grading scale: 0 = normal, 1 = mild, 2 = moderate, and 3 = severe.

Muscle atrophy and hypertrophy: Changes in the dimensions of muscle fibers and atrophic or hypertrophic properties were observed.

Muscle degeneration and congestion: Degeneration of muscle fibers and vascular congestion were observed.

Internalization of muscle nucleus and oval-central nucleus: Displacement or shape change in muscle cell nuclei were analyzed.

Fragmentation and hyalinization: Fragmentation and hyalinization were observed in muscle tissue.

Leukocyte cell infiltration: The intensity of inflammatory cell infiltration in muscle tissue was evaluated.

### 2.3. Western Blot

The Western blot method provides an analysis of the amount of a specific protein in the tissue. The amounts of target proteins cleaved caspase 3 (E-AB-30004, Elabscience, Houston, TX, USA), Bax (E-AB-13814, Elabscience, Houston, TX, USA), Bcl-2 (E-AB-64067, Elabscience, Houston, TX, USA), Nrf2 (E-AB-32280, Elabscience, Houston, TX, USA), NLRP3 (E-AB-93112, Elabscience, Houston, TX, USA), and GSDMD (AF4012, Affinity Biosciences, Cincinnati, OH, USA) were analyzed. β-actin (E-AB-40338, Elabscience, Houston, TX, USA) was used as a loading control. First of all, samples were prepared. The skeletal muscle tissue was homogenized with the RIPA solution and then centrifuged. Total protein measurement was performed in the samples using the BCA protein assay kit (23225, Thermo Fisher Scientific, Sunnyvale, CA, USA). The required volume was calculated, and the same amount of protein was loaded into the gel wells. In Western blot analysis, the separation gel and the stacking gel were prepared. Sample solutions were loaded into the gel wells and the proteins were run with the running process. Protein transfer from the gel to the membrane was performed with the transfer process and then blocking and antibody incubations were applied to the PVDF membrane. Then, the membranes were imaged with the digital imaging system. The band intensities of the target protein were calculated as a ratio to β-actin [26].

### 2.4. Enzyme-Linked Immunosorbent Assay (ELISA)

TNF-α was evaluated in supernatants obtained from rat skeletal muscle tissue homogenates. Experiments were carried out by referring to the user manual of the commercial kit (ER1393, Fine Test), and the data were measured with a spectrophotometer.

### 2.5. Oxidative Status Markers

Oxidative status markers, malondialdehyde (MDA) and glutathione (GSH) levels, and superoxide dismutase (SOD) and catalase (CAT) activities were measured in skeletal muscle tissues.

For MDA analysis, tissue samples were homogenized in cold trichloroacetic acid solution, and then the homogenates were centrifuged. Equal volumes of thiobarbituric acid and supernatants were mixed and kept in a boiling water bath at 100 °C for 15 min. The absorbance of the samples was measured at 535 nm [27].

For GSH analysis, tissue samples were homogenized in cold trichloroacetic acid solution, and then the homogenates were centrifuged. After centrifugation, the supernatant was added to the mixture of 0.3 M disodium hydrogen orthophosphate dihydrate solution and dithiobisnitrobenzoate solution. The absorbance of the samples was measured at 412 nm with a spectrophotometer [28].

Catalase activity was evaluated according to the method of Aebi et al. The method is based on the spectrophotometric determination of the amount of absorbance that changes due to the breakdown of hydrogen peroxide by catalase at a wavelength of 240 nm [29].

SOD activity is based on the principle of the formation of O_2_^−^ by xanthine with xanthine oxidase, which forms a colored compound with nitro blue tetrazolium, and the color intensity was measured with a spectrophotometer, as defined by Yi-Sun et al. The low color intensity indicates high SOD activity [30].

### 2.6. Statistical Analysis

The Statistical Package for the Social Sciences (SPSS) 24.0 program was used for statistical analysis. The conformity of the data to normal distribution was evaluated with the Shapiro–Wilk test. All data are expressed as the mean ± standard deviation (SD) or median (25–75%). Comparisons of >2 groups were performed using the Kruskal–Wallis test followed by Dunn’s post hoc test or one-way ANOVA followed by Tukey’s post hoc test. *p* < 0.05 was considered as a significant difference.

## 3. Results

### 3.1. Histopathologic Analysis

**Figure 2:**
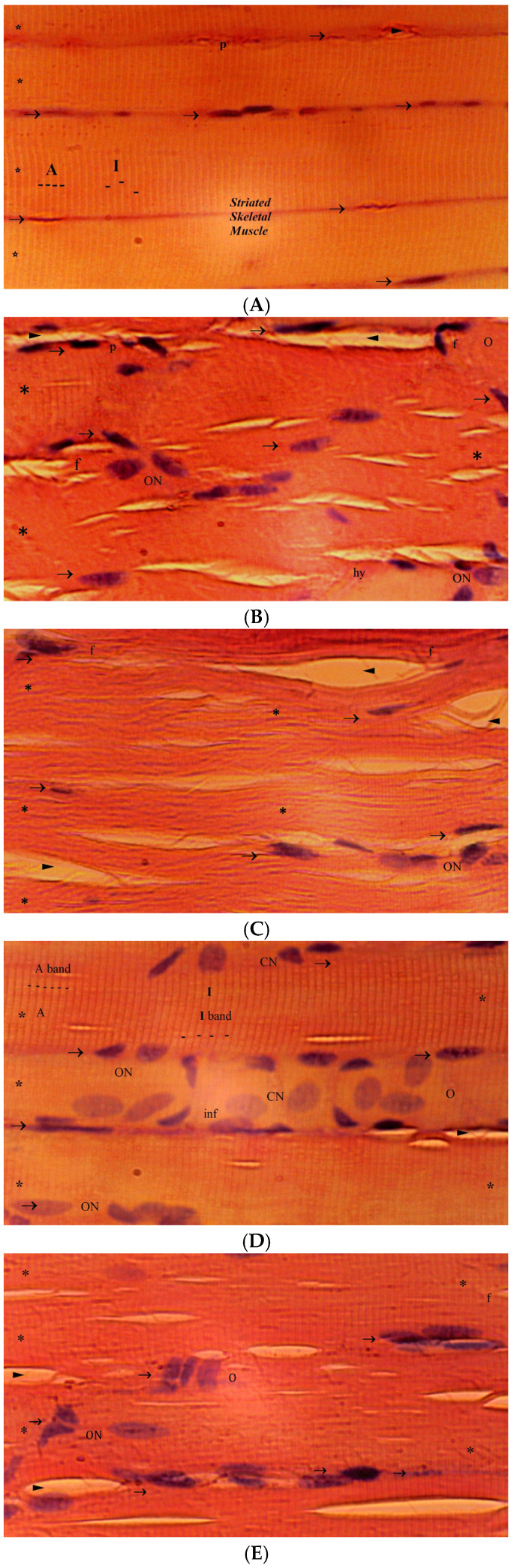
Representative images of histopathologic analysis obtained from hematoxylin and eosin staining (n = 7). (**A**): Sham group, (**B**): I/R group, (**C**): EA+I/R, (**D**): BER+I/R, and (**E**): EA/BER+I/R. A: A band, I: I band, *p*: pyknotic nucleus, *: muscle fibers, ►: intercellular space, →: peripheral flat nuclei, ON: oval nucleus, f: fragmentation, O: edema, hy: hyalinization, CN: central nucleus, and inf: infiltration.

There are significant differences between the groups in terms of muscle atrophy and hypertrophy (*p* = 0.010). In the I/R group, muscle atrophy and hypertrophy increased significantly compared to the sham group (*p* = 0.001). In the EA+I/R, BER+I/R, and EA/BER+I/R groups, muscle atrophy and hypertrophy significantly decreased compared to the I/R group (*p* = 0.016, *p* = 0.005, and *p* = 0.005, respectively) (Table 1, Figure 2). Significant differences were detected between the groups in terms of muscle degeneration and congestion (*p* = 0.009). Muscle degeneration and congestion were high in the I/R group compared to the sham group (*p* = 0.001). In the EA+I/R, BER+I/R, and EA/BER+I/R groups, muscle degeneration and congestion significantly reduced compared to the I/R group (*p* = 0.015, *p* = 0.004, and *p* = 0.004, respectively) (Table 1, Figure 2). There are significant differences between the groups in terms of internalization of the muscle nucleus and the oval-central nucleus (*p* = 0.007). In the I/R group, the internalization of the muscle nucleus and the oval-central nucleus increased according to the sham group (*p* < 0.001). In the EA+I/R, BER+I/R, and EA/BER+I/R groups, the internalization of the muscle nucleus and oval-central nucleus significantly decreased compared to the I/R group (*p* = 0.010, *p* = 0.003, and *p* = 0.003, respectively) (Table 1, Figure 2). Also, fragmentation and hyalinization were significantly different between the groups (*p* = 0.019). In the I/R group, fragmentation and hyalinization increased according to the sham group (*p* = 0.002). In the EA+I/R, BER+I/R, and EA/BER+I/R groups, fragmentation and hyalinization significantly decreased (*p* = 0.043, *p* = 0.017, *p* = 0.006, respectively) (Table 1, Figure 2). There are significant differences between the groups in terms of leukocyte cell infiltration (*p* = 0.006). In the I/R group, leukocyte cell infiltration increased according to the sham group (*p* < 0.001). In the EA+I/R, BER+I/R, and EA/BER+I/R groups, leukocyte cell infiltration significantly reduced (*p* = 0.017, *p* = 0.006, *p* = 0.002, respectively) (Table 1, Figure 2).

### 3.2. Western Blot

**Figure 3:**
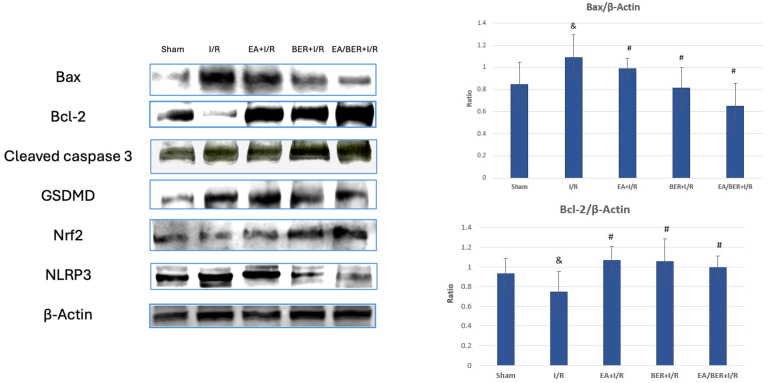

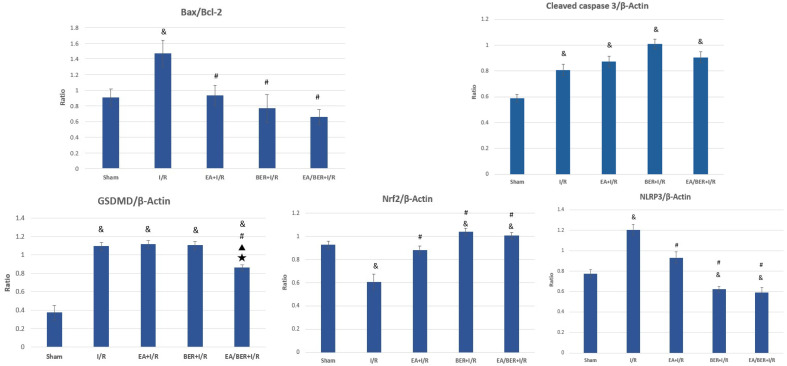
Representative images and graphs obtained from Western blot (n = 7). & *p* < 0.05 compared to the sham group, # *p* < 0.05 compared to the I/R group, ▲ *p* < 0.05 compared to the EA+I/R group, and ⋆ *p* < 0.05 compared to the BER+I/R group.

Bax/β-Actin levels were significantly high in the I/R group compared to the other groups (*p* = 0.043) (Figure 3). However, there was no statistically significant difference between the sham, EA+I/R, BER+I/R, and EA/BER+I/R groups. Bcl-2/β-Actin levels were significantly low in the I/R group compared to the other groups (*p* = 0.012) (Figure 3). Similar results were found among the sham, EA+I/R, BER+I/R, and EA/BER+I/R groups. The Bax/Bcl-2 ratio was significantly high in the I/R group compared to the other groups (*p* = 0.001), and no significant difference was found among the sham, EA+I/R, BER+I/R, and EA/BER+I/R groups. Cleaved caspase 3/β-Actin levels were significantly low in the sham group compared to the other groups (*p* = 0.001) (Figure 3). No statistically significant difference was observed among the I/R, EA+I/R, BER+I/R, and EA/BER+I/R groups. GSDMD/β-Actin levels were significantly low in the sham group compared to the other groups (*p* = 0.001) (Figure 3). The EA/BER+I/R group had lower GSDMD/β-Actin levels than the I/R group, the EA+I/R group, and the BER+I/R group (*p* = 0.001, *p* = 0.035, and *p* = 0.001, respectively). No statistical difference was found between the I/R, EA+I/R, and BER+I/R groups. Nrf2/β-Actin levels were significantly low in the I/R group compared to the other groups (*p* = 0.001) (Figure 3), while significantly high levels were observed in the BER+I/R (*p* = 0.001) and EA/BER+I/R (*p* = 0.03) groups compared to the sham group. NLRP3/β-Actin levels were significantly high in the I/R group compared to the other groups (*p* = 0.001) (Figure 3). While the levels in the sham group and the EA+I/R group were similar, significantly lower NLRP3/β-Actin levels were observed in the BER+I/R (*p* = 0.041) and EA/BER+I/R (*p* = 0.001) groups compared to the sham group.

### 3.3. Enzyme-Linked Immunosorbent Assay (ELISA)

**Figure 4:**
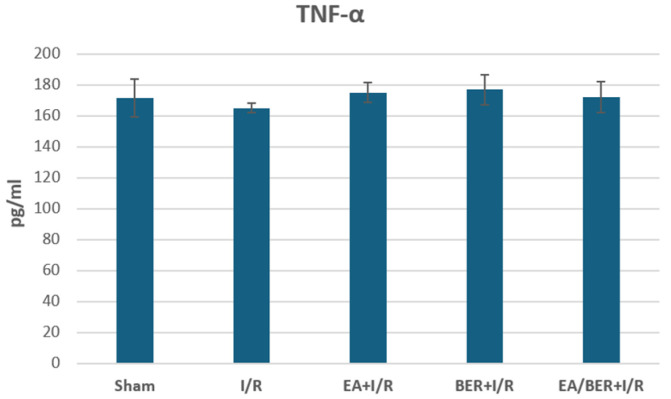
TNF-α levels obtained from ELISA (n = 7).

There was no statistical difference between the groups in terms of TNF-α levels evaluated by the ELISA method (*p* > 0.05) (Figure 4). While TNF-α levels did not change in the I/R group, no significant effects of the EA and BER treatments were observed.

### 3.4. Oxidative Status Markers

**Figure 5:**
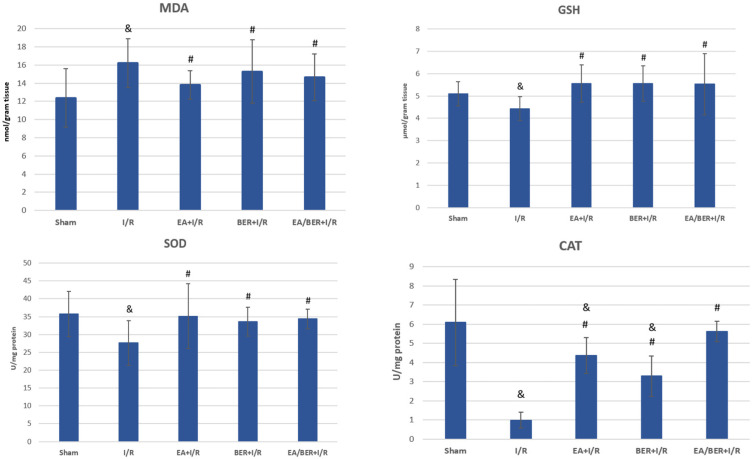
MDA, GSH, CAT, and SOD results of groups (n = 7). & *p* < 0.05 compared to the sham group; # *p* < 0.05 compared to the I/R group.

MDA levels were significantly high in the I/R group compared to the sham group (*p* = 0.023) (Figure 5). In the EA+I/R, BER+I/R, and EA/BER+I/R groups, MDA levels were similar to the sham group, and no statistical difference was observed (*p* > 0.05). GSH levels were significantly low in the I/R group compared to the other groups (*p* = 0.036) (Figure 5). The GSH levels of the sham, EA+I/R, BER+I/R, and EA/BER+I/R groups were similar, and the difference between them was not statistically significant (*p* > 0.05).

SOD activity was significantly low in the I/R group compared to the sham group (*p* = 0.018) (Figure 5). In the EA+I/R, BER+I/R, and EA/BER+I/R groups, SOD activity was similar to the sham group, and the difference between them was not statistically significant (*p* > 0.05). CAT activity was significantly low in the I/R group compared to the other groups (*p* = 0.001) (Figure 5). The EA/BER+I/R group had similar levels with the sham group (*p* > 0.05), and CAT activity was significantly low in the EA+I/R (*p* = 0.014) and BER+I/R (*p* = 0.01) groups compared to the sham group.

## 4. Discussion

Hind limb I/R injury, which has no definitive treatment and leads to serious morbidity and mortality, can cause skeletal muscle necrosis, compartment syndrome, or multiple organ failure [2]. Various agents, especially antioxidants, are being tried for treatment. Antioxidant combinations are frequently used nowadays, but whether they cause positive or negative effects is controversial. Therefore, the effects of EA and BER, whose individual antioxidant activities are well known, as well as their combination, were evaluated on hind limb I/R injury. The basis of this injury is inflammation, cell death, and oxidative stress [3,4].

I/R injury activates several pathological cascades [31]. One of them, apoptosis, is triggered by hind limb I/R injury. The levels of apoptotic markers such as Bax, Bcl-2, and cleaved caspase-3, which play an important role in the regulation of cell death, change. It is stated that hind limb I/R injury increases the expression of pro-apoptotic Bax and decreases anti-apoptotic Bcl-2, thus facilitating cellular death processes [32,33,34]. In the present study, the Bax/Bcl-2 ratio and cleaved caspase-3 levels significantly increased in the I/R groups. EA and BER significantly reduced the Bax/Bcl-2 ratio. These results demonstrate the antiapoptotic properties of EA and BER. Similarly, in a study, it was reported that EA inhibited apoptosis and reduced cellular damage in muscle tissue by reducing the Bax/Bcl-2 ratio in streptozotocin-induced diabetic muscle atrophy [35]. In a study, berberine inhibited caspase-3 expression and improved the Bcl-2/Bax ratio in hepatic I/R injury, thus protecting organ function by preventing apoptosis [36]. In diabetic rats, berberine prevented Bax-related apoptosis by increasing Bcl-2 expression and improved renal function in renal I/R injury [37]. In our study, EA and BER showed antiapoptotic effects, consistent with the literature.

The inflammatory response that occurs during I/R injury can trigger inflammation-related cell death called pyroptosis [38]. In the present study, NLRP3/β-Actin levels were significantly high in the I/R group compared to the other groups. These results suggest that pyroptosis-related inflammasome formation is induced by hind limb I/R injury. In addition, GSDMD/β-Actin levels increased in the I/R group compared to the sham group, while they decreased in the EA and BER combination group. As a result of these data, we conclude that EA and BER may have antipyroptotic effects. In a study, EA inhibited inflammation and cell death via antipyroptotic effects on GSDMD and NLRP3 inflammasomes in hepatic I/R injury. Thus, it was emphasized that EA exhibited organ-protective effects by suppressing pyroptosis [39]. Similarly, the suppressive effects of berberine on the NLRP3 inflammasome and GSDMD have been demonstrated in diabetic cardiomyopathy [40]. The findings of our study also suggest that the combination of EA and BER has potential effects to reduce pyroptosis in hind limb I/R injury.

TNF-α has pleiotropic effects. Different effects of TNF-α on different cells are observed at different stages of inflammation. It has been reported that while it activates the body’s defense against infections in low concentrations, it causes inflammation and tissue damage in high concentrations [41]. Therefore, the TNF-α levels in the present study may be related to the model and duration of the study.

During I/R injury, Nrf2 plays a critical role in the prevention of oxidative stress [42]. In our study, it was observed that the combination of EA and BER significantly increased the Nrf2/β-Actin levels, which were decreased in the I/R group. According to these results, EA and BER also have ameliorative effects via increasing Nrf2 levels. Aslan et al. showed that EA prevented kidney damage, reduced lipid peroxidation, and increased antioxidant enzyme activities by activating the Nrf2 pathway in a carbon tetrachloride-induced oxidative stress model [43]. Altamimi et al. demonstrated that EA prevented kidney injury, inflammation, and oxidative stress in a STZ-induced diabetic nephropathy model by enhancing the transcriptional activation of Nrf2 and reducing its interaction with Keap1 [44]. In another study, it was shown that BER increased the activation of the Nrf2/ARE signaling pathway and reduced oxidative stress and suppressed lipid accumulation in liver tissue [45]. BER prevented neuronal apoptosis by activating the ERK/Nrf2 signaling pathway, increased the expression of antioxidant genes such as HO-1, and supported neuronal survival by reducing neuronal loss in the brain in Wang et al.’s study [46]. In our study, we concluded that the combination of EA and BER may exhibit tissue-protective effects on hind limb I/R injury via activating the Nrf2 pathway, similar to previous studies.

Hind limb I/R injury also induces an inflammatory reaction, resulting in the formation of ROS, which increases local tissue damage [47,48]. It is known that ROS have harmful effects on lipid structures in cell membranes, intracellular structural and functional proteins, and genetic material, leading to cell damage or death [49]. Various studies have shown that hind limb I/R injury suppresses antioxidants such as SOD, GSH, CAT, and glutathione peroxidase and causes tissue damage by increasing MDA levels, the end product of lipid peroxidation [50,51]. In a study, the effects of berberine and coenzyme Q10 on hind limb I/R injury were evaluated, and the increased MDA and myeloperoxidase in the ischemia group were reduced by berberine supplementation. GSH, SOD, and CAT, which were decreased by the ischemia procedure, were increased by berberine. Berberine was reported to protect against oxidative damage on hind limb I/R injury [52]. Consistent with the literature, in our study, MDA levels, an indicator of cell membrane lipid damage, were highest in the I/R group. The decrease in MDA levels with EA and BER shows the protective effect of these antioxidants. Similarly, the increase in the SOD, CAT, and GSH can be considered as the ameliorative effects of EA and BER on hind limb I/R injury.

The present study has some limitations. In this study, EA and BER were given in a single dose, and their effects were investigated during the I/R procedure. Our future scope is to evaluate the efficacy of long-term supplementation of EA, BER, and combined treatment.

## 5. Conclusions

As a result, EA and BER have shown alleviating effects on hind limb I/R injury with their antiapoptotic, antipyroptotic, and antioxidant properties. The combination of EA and BER was found to be more effective on hind limb I/R injury in terms of reducing the histopathological parameters of fragmentation–hyalinization and leukocyte cell infiltration, decreasing Bax and Gasdermin D levels and increasing CAT activity more effectively. Therefore, it is concluded that the combination of EA and BER may be a potential treatment option on hind limb I/R injury.

## Figures and Tables

**Table 1:** Gastrocnemius muscle tissue results obtained from H&E staining [Median (25–75%)].

	Sham (n = 7)	I/R (n = 7)	EA+I/R (n = 7)	BER+I/R (n = 7)	EA/BER+I/R (n = 7)	*p* **
Muscle atrophy–hypertrophy	0.00 (0–1)	1.50 (1–2) ^&^	1.00 (0–1) ^#^	0.50 (0–1) ^#^	0.50 (0–1) ^#^	0.010
Muscle degeneration–congestion	0.00 (0–1)	1.00 (1–2) ^&^	0.00 (0–1) ^#^	0.00 (0–1) ^#^	0.00 (0–1) ^#^	0.009
Internalization of muscle nucleus–oval-central nucleus	0.00 (0–1)	1.00 (0–1) ^&^	0.00 (0–1) ^#^	0.00 (0–1) ^#^	0.00 (0–1) ^#^	0.007
Fragmentation–hyalinization	0.00 (0–1)	1.50(0–1) ^&^	1.00 (0–1) ^#^	0.50 (0–1) ^#^	0.00 (0–1) ^#^	0.019
Leukocyte cell infiltration	0.00 (0–1)	1.50 (1–2) ^&^	1.00 (0–1) ^#^	0.50 (0–1) ^#^	0.00 (0–1) ^#^	0.006

** *p* < 0.05: significance level with Kruskal–Wallis test. ^&^ *p* < 0.05: significant difference compared to sham group. ^#^ *p* < 0.05: significant difference compared to I/R group.

## Data Availability

The original contributions presented in this study are included in the article. Further inquiries can be directed to the corresponding author.

## References

[1] Ljungman C., Adami H.O., Bergqvist D., Sparen P., Bergström R. (1991). Risk factors for early lower limb loss after embolectomy for acute arterial occlusion: A population-based case-control study. Br. J. Surg..

[2] Björck M., Earnshaw J.J., Acosta S., Bastos Gonçalves F., Cochennec F., Debus E.S., Hinchliffe R., Jongkind V., Koelemay M.J.W., Menyhei G. (2020). Editor’s Choice—European Society for Vascular Surgery (ESVS) 2020 Clinical Practice Guidelines on the Management of Acute Limb Ischaemia. Eur. J. Vasc. Endovasc. Surg..

[3] Sies H., Berndt C., Jones D.P. (2017). Oxidative stress. Annu. Rev. Biochem..

[4] Wu L., Xiong X., Wu X., Ye Y., Jian Z., Zhi Z., Gu L. (2020). Targeting oxidative stress and inflammation to prevent ischemia-reperfusion injury. Front. Mol. Neurosci..

[5] Grilo A.L., Mantalaris A. (2019). Apoptosis: A mammalian cell bioprocessing perspective. Biotechnol. Adv..

[6] Al-Salam S., Hashmi S. (2018). Myocardial ischemia-reperfusion injury: Apoptotic, inflammatory and oxidative stress role of Galectin-3. Cell Physiol. Biochem..

[7] Asadi M., Taghizadeh S., Kaviani E., Vakili O., Taheri-Anganeh M., Tahamtan M., Savardashtaki A. (2022). Caspase-3: Structure, function, and biotechnological aspects. Biotechnol. Appl. Biochem..

[8] Edlich F. (2018). BCL-2 proteins and apoptosis: Recent insights and unknowns. Biochem. Biophys. Res. Commun..

[9] Liu Y., Zhang J., Zhang D., Yu P., Zhang J., Yu S. (2022). Research progress on the role of pyroptosis in myocardial ischemia-reperfusion injury. Cells.

[10] Del Re D.P., Amgalan D., Linkermann A., Liu Q., Kitsis R.N. (2019). Fundamental mechanisms of regulated cell death and implications for heart disease. Physiol. Rev..

[11] Bardallo R.G., Panisello-Roselló A., Sanchez-Nuno S., Alva N., Roselló-Catafau J., Carbonell T. (2022). Nrf2 and oxidative stress in liver ischemia/reperfusion injury. FEBS J..

[12] Derosa G., Maffioli P., Sahebkar A. (2016). Ellagic acid and its role in chronic diseases. Advances in Experimental Medicine and Biology.

[13] Durgun C., Aşir F. (2022). Effect of ellagic acid on damage caused by hepatic ischemia reperfusion in rats. Eur. Rev. Med. Pharmacol. Sci..

[14] Liu Q., Liang X., Liang M., Qin R., Qin F., Wang X. (2020). Ellagic acid ameliorates renal ischemic-reperfusion injury through NOX4/JAK/STAT signaling pathway. Inflammation.

[15] Şekerci Ç.A., Aydın H.R., Livaoğlu A. (2023). Protective effects of ellagic acid on testicular ischemia-reperfusion injury in rats. J. Urol. Surg..

[16] Hassonizadeh Falahieh K., Sarkaki A., Edalatmanesh M., Gharib Naseri M.K., Farbood Y. (2020). Ellagic acid attenuates post-cerebral ischemia and reperfusion behavioral deficits by decreasing brain tissue inflammation in rats. Iran. J. Basic Med. Sci..

[17] Kumar A., Ekavali, Chopra K., Mukherjee M., Pottabathini R., Dhull D.K. (2015). Current knowledge and pharmacological profile of berberine: An update. Eur. J. Pharmacol..

[18] Huang Z., Han Z., Ye B., Dai Z., Shan P., Lu Z., Dai K., Wang C., Huang W. (2015). Berberine alleviates cardiac ischemia/reperfusion injury by inhibiting excessive autophagy in cardiomyocytes. Eur. J. Pharmacol..

[19] Zhao G.L., Yu L.M., Gao W.L., Duan W.X., Jiang B., Liu X.D., Zhang B., Liu Z.H., Zhai M.E., Jin Z.X. (2016). Berberine protects rat heart from ischemia/reperfusion injury via activating JAK2/STAT3 signaling and attenuating endoplasmic reticulum stress. Acta Pharmacol. Sin..

[20] Liu H., Ren X., Ma C. (2018). Effect of berberine on angiogenesis and HIF-1α/VEGF signal transduction pathway in rats with cerebral ischemia-reperfusion injury. J. Coll. Phys. Surg. Pak..

[21] Zheng H., Lan J., Li J., Lv L. (2018). Therapeutic effect of berberine on renal ischemia-reperfusion injury in rats and its effect on Bax and Bcl-2. Exp. Ther. Med..

[22] Assaran A.H., Akbarian M., Amirahmadi S., Salmani H., Shirzad S., Hosseini M., Beheshti F., Rajabian A. (2022). Ellagic Acid Prevents Oxidative Stress and Memory Deficits in a Rat Model of Scopolamine-induced Alzheimer’s Disease. Cent. Nerv. Syst. Agents Med. Chem..

[23] Saha L., Kumari P., Rawat K., Gautam V., Sandhu A., Singh N., Bhatia A., Bhattacharya S., Sinha V.R., Chakrabarti A. (2023). Neuroprotective effect of Berberine Nanoparticles Against Seizures in Pentylenetetrazole Induced Kindling Model of Epileptogenesis: Role of Anti-Oxidative, Anti-Inflammatory, and Anti-Apoptotic Mechanisms. Neurochem. Res..

[24] Lei F., Xing D.M., Xiang L., Zhao Y.N., Wang W., Zhang L.J., Du L.J. (2003). Pharmacokinetic study of ellagic acid in rat after oral administration of pomegranate leaf extract. J. Chromatogr. B Anal. Technol. Biomed. Life Sci..

[25] Feng X., Wang K., Cao S., Ding L., Qiu F. (2021). Pharmacokinetics and Excretion of Berberine and Its Nine Metabolites in Rats. Front. Pharmacol..

[26] Mahmood T., Yang P.C. (2012). Western blot: Technique, theory, and troubleshooting. N. Am. J. Med. Sci..

[27] Casini A.F., Ferrali M., Pompella A., Maellaro E., Comporti M. (1986). Lipid peroxidation and cellular damage in extrahepatic tissues of bromobenzene-intoxicated mice. Am. J. Pathol..

[28] Aykaç G., Uysal M., Yalçin A.S., Koçak-Toker N., Sivas A., Oz H. (1985). The effect of chronic ethanol ingestion on hepatic lipid peroxide, glutathione, glutathione peroxidase, and glutathione transferase in rats. Toxicology.

[29] Aebi H., Suter H., Feinstein R.N. (1968). Activity and stability of catalase in blood and tissues of normal and acatalasemic mice. Biochem. Genet..

[30] Sun Y., Oberley L.W., Li Y. (1988). A simple method for clinical assay of superoxide dismutase. Clin. Chem..

[31] Yadava S., Reddy D.H., Nakka V.P., Anusha V.L., Dumala N., Viswanadh M.K., Chakravarthi G., Nalluri B.N., Ramakrishna K. (2025). Unravelling neuroregenerative and neuroprotective roles of Wnt/β-catenin pathway in ischemic stroke: Insights into molecular mechanisms. Neuroscience.

[32] Zhao D., Zhang M., Yuan H., Meng C., Zhang B., Wu H. (2018). Ginsenoside Rb1 protects against spinal cord ischemia-reperfusion injury in rats by downregulating the Bax/Bcl-2 ratio and caspase-3 and p-Ask-1 levels. Exp. Mol. Pathol..

[33] Yaidikar L., Thakur S. (2015). Punicalagin attenuated cerebral ischemia-reperfusion insult via inhibition of proinflammatory cytokines, up-regulation of Bcl-2, down-regulation of Bax, and caspase-3. Mol. Cell. Biochem..

[34] Yuan T., Yang N., Bi W., Zhang J., Li X., Shi L., Liu Y., Gao X. (2021). Protective role of sulodexide on renal injury induced by limb ischemia-reperfusion. Evid. Based Complement. Altern. Med..

[35] Liu X., Cheng C., Deng B., Liu M. (2022). Ellagic acid attenuates muscle atrophy in STZ-induced diabetic mice. Physiol. Res..

[36] Sheng M., Zhou Y., Yu W., Weng Y., Xu R., Du H. (2015). Protective effect of berberine pretreatment in hepatic ischemia/reperfusion injury of rat. Transplant. Proc..

[37] Kumaş M., Eşrefoğlu M., Karataş E., Duymaç N., Kanbay S., Ergün I.S., Üyüklü M., Koçyiğit A. (2019). Investigation of dose-dependent effects of berberine against renal ischemia/reperfusion injury in experimental diabetic rats. Nefrología.

[38] Zheng Y., Xu X., Chi F., Cong N. (2022). Pyroptosis: A newly discovered therapeutic target for ischemia-reperfusion injury. Biomolecules.

[39] Wang H., Miao F., Ning D., Shan C. (2022). Ellagic acid alleviates hepatic ischemia-reperfusion injury in C57 mice via the Caspase-1-GSDMD pathway. BMC Vet. Res..

[40] Zhong C., Xie Y., Wang H., Chen W., Yang Z., Zhang L., Deng Q., Cheng T., Li M., Ju J. (2024). Berberine inhibits NLRP3 inflammasome activation by regulating mTOR/mtROS axis to alleviate diabetic cardiomyopathy. Eur. J. Pharmacol..

[41] Kalliolias G.D., Ivashkiv L.B. (2016). TNF biology, pathogenic mechanisms and emerging therapeutic strategies. Nat. Rev. Rheumatol..

[42] Sun Y., Yang X., Xu L., Jia M., Zhang L., Li P., Yang P. (2023). The role of Nrf2 in relieving cerebral ischemia-reperfusion injury. Curr. Neuropharmacol..

[43] Aslan A., Gok O., Beyaz S., Ağca C.A., Erman O., Zerek A. (2020). Ellagic acid prevents kidney injury and oxidative damage via regulation of Nrf-2/NF-κB signaling in carbon tetrachloride-induced rats. Mol. Biol. Rep..

[44] Altamimi J.Z., Alfaris N.A., Alshammari G.M., Alagal R.I., Aljabryn D.H., Aldera H., Alkhateeb M.A., Yahya M.A. (2020). Ellagic acid protects against diabetic cardiomyopathy in rats by stimulating cardiac silent information regulator 1 signaling. J. Physiol. Pharmacol..

[45] Deng Y., Tang K., Chen R., Nie H., Liang S., Zhang J., Zhang Y., Yang Q. (2019). Berberine attenuates hepatic oxidative stress in rats with non-alcoholic fatty liver disease via the Nrf2/ARE signaling pathway. Exp. Ther. Med..

[46] Wang N., Tian Y., Yan F., Zhao F., Wang R., Luo Y., Zheng Y. (2022). Berberine protects against chronic cerebral hypoperfusion-induced cognitive impairment and hippocampal damage via regulation of the ERK/Nrf2 pathway. J. Chem. Neuroanat..

[47] Liu Z., Qu M., Yu L., Song P., Chang Y. (2018). Artesunate inhibits renal ischemia-reperfusion-mediated remote lung inflammation through attenuating ROS-induced activation of NLRP3 inflammasome. Inflammation.

[48] Gao S., Zhan L., Yang Z., Shi R., Li H., Xia Z., Yuan S., Wu Q.P., Wang T., Yao S. (2017). Remote Limb Ischaemic Postconditioning Protects Against Myocardial Ischaemia/Reperfusion Injury in Mice: Activation of JAK/STAT3-Mediated Nrf2-Antioxidant Signalling. Cell. Physiol. Biochem. Int. J. Exp. Cell. Physiol. Biochem. Pharmacol..

[49] Zuo L., Zhou T., Pannell B.K., Ziegler A.C., Best T.M. (2015). Biological and physiological role of reactive oxygen species—The good, the bad and the ugly. Acta Physiol..

[50] Gürsul C., Ekinci Akdemir F.N., Akkoyun T., Can İ., Gül M., Gülçin İ. (2016). Protective effect of naringin on experimental hindlimb ischemia/reperfusion injury in rats. J. Enzym. Inhib. Med. Chem..

[51] Ashrafzadeh Takhtfooladi M., Ashrafzadeh Takhtfooladi H., Sedaghatfar H., Shabani S. (2015). Effect of low-level laser therapy on lung injury induced by hindlimb ischemia/reperfusion in rats. Lasers Med. Sci..

[52] Apaydin Yildirim B., Annour Adoum B. (2019). The investigation of the preventive effects of Coenzyme Q10 and Berberine for tourniquet induced ischemia-reperfusion injury on skeletal muscle in rat hindlimb. GSC Biol. Pharm. Sci..

